# Hospital library closures and consolidations: a case series

**DOI:** 10.5195/jmla.2019.520

**Published:** 2019-04-01

**Authors:** Andrea Harrow, Lisa A. Marks, Debra Schneider, Alexander Lyubechansky, Ellen Aaronson, Lynn Kysh, Molly Harrington

**Affiliations:** Manager, Hospital Library, Good Samaritan Hospital, Los Angeles, CA, library@goodsam.org; Director of Libraries, Mayo Clinic Library, Mayo Clinic Scottsdale, Scottsdale, AZ, marks.lisa@mayo.edu; Supervisor, Libraries and Continuing Medical Education, HonorHealth Scottsdale Osborn Medical Center, HonorHealth System, Scottsdale, AZ, Debra.Schneider@HonorHealth.com; Assistant Professor, Clinical Librarian, Savitt Medical Library, University of Nevada, Reno, NV, alexl@med.unr.edu; Librarian, Mayo Clinic Libraries, Mayo Clinic, Rochester, MN, ellen.aaronson@gmail.com; Clinical & Research Librarian, Norris Medical Library, University of Southern California and Children’s Hospital Los Angeles, Los Angeles, CA, lkysh@chla.usc.edu; Manager, Health Sciences Library, Dignity Health St. Joseph’s Hospital & Medical Center, Phoenix, AZ, Molly.Harrington@DignityHealth.org

## Abstract

**Background:**

Health sciences libraries are being closed or are under threat of closure, but little is published that looks at context and causes or alternative library service delivery models such as affiliations or consolidations. There is also very little research about the effect of these changes on health care provider satisfaction, patient care, or hospital quality indicators. Preventing library closures is not always possible, but understanding some of the circumstances leading to the decision and implementation of a closure or consolidation could inform best practice management.

**Case Presentations:**

At a recent Medical Library Association joint chapter meeting, a panel of six librarians presented their cases of navigating a library closure or reorganization. Background information was given to highlight reasons that the decisions to reorganize or close were made. Following the case presentations, participants took part in discussion with audience members. Cases and discussion points were recorded for further research, publication, and advocacy.

**Conclusions:**

Several points from the cases are highlighted in the discussion section of the paper. An accurate reporting of US health sciences libraries and librarian staffing is needed. More needs to be written about new library service models and best practices for centralizing and maintaining library services. After a consolidation, remaining librarians will be expected to manage the effects of staff loss and site closures and so should be involved in planning and implementing these decisions. It remains to be determined how hospitals with librarians compare in patient care and other quality indicators against hospitals without librarians.

## INTRODUCTION

Hospital library closures and their effects on patients and health care providers have been cause for concern [1–3], leading to increasing publications about library mergers and changing service models [4–6]. In 2005, Medical Library Association (MLA) Past President M.J. Tooey, AHIP, FMLA, convened the MLA Vital Pathways: The Hospital Libraries Project to gather data on the status of hospital libraries. In her words, “The project quickly grew beyond the need for a survey to a more philosophical one focused on the future of hospital librarians” [7]. Indeed, while the Vital Pathways project presented data about library closures, it also focused on future roles for librarians and advocacy for librarian involvement in accreditation standards [8, 9]. The project’s work showed that the percentage of hospitals with libraries declined from around 44% in 1989 to around 30% in 2006 due to financial constraints and changes in technology [10].

More recent data also showed a decline in medical libraries. The *Library and Book Trade Almanac, 2007*, reported the existence of 2,055 US medical libraries [11], while the 2017 edition reported only 1,384 [12], a further 30% decrease over the last 10 years. DOCLINE library reports showed a similar 30% decrease: 3,166 libraries were registered in 2007 and 2,140 in 2017; though this could partly be due to shrinking collections, perceived license restrictions, lack of staff to manage interlibrary loans (ILLs), or reasons other than closure or consolidation [13]. This leads to several questions: What does this decline in hospital libraries indicate? Are the remaining libraries staffed by librarians? How do hospital libraries with and without librarians compare in provider satisfaction, patient care, and other health care quality indicators? What are new and future models of consolidated or consultant librarian services, and how successful might they be?

Librarians in the MLA Hospital Libraries Section (HLS) have been discussing the alarming trend in closing libraries and the lack of associated research and literature. Several HLS members collaborated on developing a special session panel discussion on this topic for the joint chapter meeting of the Medical Library Group of Southern California and Arizona and the Northern California and Nevada Medical Library Group that was held in Scottsdale, Arizona, in January 2018. In keeping with the conference theme, the session was called “Navigating the Waters of Hospital Library Mergers, Crossing the Canyon of Hospital Library Closures: An M&M (Mortality & Mergers) Case Conference.” Similar to a clinical case conference, six librarians each had five minutes to present their cases and give some background about what led to the decisions to close or consolidate their libraries, talk about their roles in the process, and discuss how their hospital patrons or communities were affected (supplemental Appendix). They were also asked to discuss how the process could have been handled differently and to share advice for other librarians in a similar situation. After the cases were presented, time was allowed for audience questions and group discussion.

The panelists’ aim was to identify how and why a hospital library might close or merge by looking for common factors that gave rise to these decisions. We also wanted to determine how patrons were affected and establish best practices or identify potential pitfalls for librarians on a similar trajectory. Giving voice to the librarians who navigated these difficult times was also part of our approach. Some of the cases are edited here for readability and brevity.

## CASE PRESENTATIONS

### Library closure: West Hills Hospital & Medical Center

#### Ellen Aaronson, MLS, AHIP

West Hills Hospital & Medical Center, in a suburb of Los Angeles, is part of the Healthcare Corporation of America (HCA), a for-profit hospital system of over 160 hospitals. Toward the middle of the 2000s, several changes occurred at West Hills that had a definite impact on the library services. Technology continued to advance, and the younger generations of health care providers began receiving training in online resources in medical school, thus enabling them to do their own clinical searching. Little clinical research is done in small community hospitals, so my user statistics began to decline. In order to keep myself involved and to justify my existence, I began assisting the continuing medical education (CME) coordinator with the program accreditation process. I also assisted the Medical Staff Services Department in updating physician privileges and analyzing admissions data for creating physician report cards.

Medical staff physician demographics also began to change. Los Angeles is home to several large medical centers and medical school campuses. These medical centers have opened satellite facilities in close proximity to small hospitals and employ practitioners who have applied for and received privileges at the small hospitals. These physicians have access to the information resources at their parent institutions and, therefore, have no need for those offered by the hospital library.

While the HCA librarians were successful in creating a fantastic platform of shared resources, this development also created the downward spiral for my hospital and several others. Teaching hospitals in the system have educational and training support requirements through the Accreditation Council for Graduate Medical Education, but West Hills did not fall into that category. Thus, the feeling was that physicians could simply utilize the HCA corporate resources and did not need the assistance of a library or librarian.

By 2017, my statistics had plummeted, and it was a daily struggle to remain afloat. I polished my elevator speech, spoke with the hospital administration, enlisted help from colleagues, and cited articles from MLA about the value of hospital libraries and libraries but was notified that my position and the library would no longer be supported.

I received an amazing outpouring of support from the library world following the announcement about the elimination of my position and the library. Medical librarians—those in hospitals, academia, and the ranks of MLA—began to once again discuss hospital library closures and the need for action. HLS is continuing to actively discuss how librarians should be assessing our value and is considering reinitiating its Standards Committee. In 2002 and again in 2008, MLA published “Standards for Hospital Libraries,” a guideline to ensure that hospitals have the resources and services to effectively meet their needs for knowledge-based information. Given the advancement of technology and changing times, a review of these standards is warranted.

Unfortunately in my situation, the bottom line was money. Hospital libraries are not revenue-producing departments, and regardless of our accomplishments and commitment to patient care, they are seen as expendable when budgets are reviewed.

### Library merger: Dignity Health

#### Molly Harrington, MLS

In July 2000, when I first started working at St. Joseph’s Hospital in Phoenix, there were twenty-five libraries in the Dignity Health system, which consisted of approximately thirty-eight hospitals (then called Catholic Healthcare West). Every library had at least one employee, and most of those libraries had a full-time, degreed librarian on staff.

As time went on and librarians quit, retired, or were reassigned to another department, those positions were often eliminated, and the libraries either remained open with only clerical staff or were closed. Literature searching requests, provision of articles, and ILL requests were sometimes picked up by other Dignity Health hospitals, but not always.

Today, the Dignity Health system comprises 39 acute care hospitals and 250 ancillary care sites, which include more than 60,000 employees and medical staff. Library services are being provided by a total of 6 libraries with 9 library employees who total 6 full-time equivalents (FTEs). Only 4 of the 9 library staff are degreed librarians, 2 of whom are at St. Joseph’s, meaning that only 3 Dignity Health hospitals have degreed librarians on staff. Current library staff provides services to 18 Dignity Health hospitals, including 9 hospitals with residency programs, which leaves more than half of the Dignity Health hospitals without consistent library services. Hospitals that are not served by a Dignity Health library have access to the online resources that are provided across the system.

St. Joseph’s is by far the largest hospital in the Dignity Health system, with 595 beds, over 4,000 FTEs, over 1,000 medical staff, 9 residency programs, 7 fellowship programs, and a large research department with over 100 scientists. As such, it makes sense that the library at St. Joseph’s takes the lead in organizing and directing the delivery of library services consistently throughout the system. The current “model” for providing library services to all Dignity Health hospitals has been by default. In other words, if a Dignity Health staff member has a library need and they do not have a librarian on site, they tend to contact the first Dignity Health library for which they can find contact information.

It was recently announced that the librarian position at St. Mary’s Long Beach, California, would be kept and the library would remain open after their longtime librarian retired last December. It was also recently announced that Dignity Health and Catholic Health Initiatives are to partner and create a new nonprofit health system that will integrate more than 130 hospitals and 700 care sites across 28 states, with 159,000 employees and 25,000 physicians and other advanced-practice clinicians. How this will affect the libraries is currently unknown.

### University and hospital collaboration: University of Southern California and Children’s Hospital Los Angeles

#### Lynn Kysh, MLIS

Children’s Hospital Los Angeles (CHLA) has been affiliated with the University of Southern California (USC) since 1932, with physician leaders holding faculty appointments in the university’s Department of Pediatrics. In 1999, a medical library management service agreement was made between the USC Health Sciences Libraries and CHLA’s Academic Affairs Department (Table 1). The goal of this agreement was to ensure the delivery of library services in the hospital through collaboration between the university and the hospital. The university would supply and manage a librarian who would work on-site half the work week, while the hospital covered the cost of and ensured space and support.

**Table 1:**
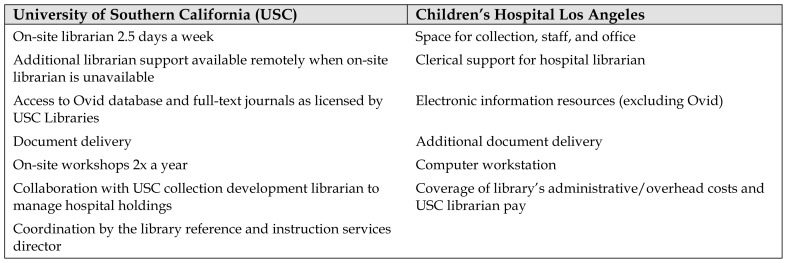
Services provided by the University of Southern California and Children’s Hospital Los Angeles in the medical library management service agreement (1999)

University of Southern California (USC)	Children’s Hospital Los Angeles
On-site librarian 2.5 days a week	Space for collection, staff, and office
Additional librarian support available remotely when on-site librarian is unavailable	Clerical support for hospital librarian
Access to Ovid database and full-text journals as licensed by USC Libraries	Electronic information resources (excluding Ovid)
Document delivery	Additional document delivery
On-site workshops 2x a year	Computer workstation
Collaboration with USC collection development librarian to manage hospital holdings	Coverage of library’s administrative/overhead costs and USC librarian pay
Coordination by the library reference and instruction services director	

The contract is still in place, and I was hired as the new librarian when my predecessor left in 2015. When the physical space of the library closed in 2013, the holdings became completely electronic. As the librarian, I work out of the hospital family resource center, which is a centralized and easy-to-find location but is susceptible to noise and conflicting uses, as it functions primarily as a space for patients and their families.

A persistent dilemma that is yet to be resolved through collaboration is equal access to information. Currently, physicians are affiliated with the university as faculty, residents, interns, or fellows and have full access to the university’s resources, which are significantly greater than those available to other health care providers, who are limited to hospital resources. However, this dilemma is an opportunity for library services to support interprofessionalism, patient-centered care, and evidence-based practice. Collaboration between the faculty physicians, librarian, and other health care providers creates a shared learning environment that results in more confident decision-making, greater communication, and better patient care.

### Cancelled cooperative agreement: University Medical Center of Southern Nevada, Las Vegas, and University of Nevada, Reno, School of Medicine

#### Alexander Lyubechansky, MA, MLIS

In 2008, the hospital known as University Medical Center (UMC) of Southern Nevada was experiencing financial challenges. To save money, UMC was considering closing down its well-established medical library that had served the medical community of Las Vegas since the early 1960s. The University of Nevada, Reno (UNR), School of Medicine had many residents rotating at UMC and offered assistance in keeping the library open. As a result, a cooperative agreement was negotiated and signed. It stipulated that the UNR School of Medicine would provide a 0.75-FTE professional librarian to provide document delivery, reference, instruction, and other library services, including negotiating licensing agreements for hospital information resources. Work stations in the library would be divided between two networks, with UMC providing space, operating support, and financial support.

In May 2011, I was hired as the clinical librarian for the UMC, filling the vacancy for this solo librarian position. I became faculty of the UNR School of Medicine as well as a contractor for UMC. In addition to serving UNR School of Medicine residency and clerkship programs, I quickly became engaged in hospital-related activities, widely promoted library services to the hospital community, and created an information literacy program with several accredited sessions for nurses and allied health professionals. From 2011 to 2015, library business thrived as I continued to serve the information needs of the hospital as well as the wider medical community of Las Vegas.

Throughout that period, UMC was going through financial and organizational changes, with three chief executive officers (CEOs) rotating through in a short period of time. Meanwhile, in 2014–2015, it became known that the University of Nevada Las Vegas (UNLV) would be establishing a new medical school and that UMC would become its partner. In 2015, UMC decided to terminate the library services cooperative agreement with UNR. Savitt Medical Library, UNR, where I was faculty, proposed several plans to UMC to preserve essential library services. However, the proposals were not accepted, and on July 1, 2015, the UMC Medical Library ceased to exist. To ensure uninterrupted support for UNR School of Medicine students, residents, and faculty, Savitt Medical Library established a branch medical library in a location close to UMC, and I relocated there.

During the transitional period prior to the UMC Medical Library’s final closing, Savitt Medical Library made suggestions to UMC regarding maintaining the library website but never received an answer. Moreover, it appeared that the vast majority of hospital employees were not notified about the library closure, and they continued to seek my help. My new location and contact information was passed by word of mouth, and numerous nurses continued to seek my help with their research and study needs.

Information that I received from some UMC employees indicated that two or three online library resources continued to be available. However, not everyone was aware of them. More than two years later, I still occasionally receive requests from UMC staff.

I think this closure of the library could have been handled differently. Savitt Medical Library, UNR, offered to help UMC offset the negative impact of the library closure, but the offers were not answered. Also, UMC could have solicited their employees’ opinions and informed their employees about the library closing in a more straightforward manner.

In 2016, the new UNLV School of Medicine was established, and its new Health Sciences Library operates out of the same space as the former UMC Medical Library. At this point, I am not aware of plans for the UNLV Health Sciences Library to extend its services to the UMC community.

### Library merger: Providence Health System

#### Lisa Marks, MLS, AHIP

At my former institution, Providence Saint Joseph Medical Center, Burbank, California, a new administrative team was brought on board in 2012. I was worried about this new team and wondered about their thoughts on library services. As time went on and the new team settled in, the new CEO and chief administrative officer would periodically wander through the library. Initially, I believed they were looking at the space the library occupied and feared they wanted to downsize the library space. However, that was not the case, and in hindsight, I believe they were looking to see how much the library was used and who was using it. I also got the impression that this new CEO was a “cleaner,” meaning he was going to come in, clean house, and leave. That is exactly what happened.

Library staff included a 1.0 FTE library manager position and 1.5 FTE paraprofessional positions. In June 2013, as we were trying to regionalize our services for the 4 Providence hospitals in Southern California, we heard rumors of future layoffs. My manager and I expected that the paraprofessional staff of 1.5 FTEs would be the ones let go. However, both my manager and I were shocked to learn that the positions being eliminated from the library staff were a 0.5-FTE paraprofessional position as well as my 1.0 FTE library manager position, leaving a 1.0 FTE paraprofessional to run the library. The library was left in capable hands, as the paraprofessional had a master of library and information science (MLIS) degree and could handle the librarian duties. Knowing about the paraprofessional’s credentials, on my departure and final meeting with the Human Resources (HR) office, I asked the HR representative to promote the paraprofessional to librarian, as had been previously requested. Unfortunately, the promotion was not given, and by September 2013, the paraprofessional left in pursuit of a professional position, leaving the library unstaffed. The library did not close, however, and by 2015, the hospital realized that it did indeed need a librarian. A new position was created and rolled into the now system-wide library services department for Providence Health & Services.

Once my shock, dismay, and anger subsided, I was able to focus on finding a new position. Although it took seven months, I am now thrilled to be working for a world-renowned medical institution focused on patient care, education, and research.

### System merger and circuit librarian pilot: HonorHealth System

#### Debra Schneider, MLIS, MEd

In 2013, it was announced that Scottsdale Healthcare (SHC) would affiliate and merge with John C. Lincoln (JCL), another local hospital system. The plans were to merge into a five-campus teaching hospital system with local oversight and leadership. Because both organizations already had library services, we were asked to create a business plan describing how we would standardize library services and bring cost savings to the new organization.

The two systems had very different library service models. The SHC side had very robust library services with five librarians, two technicians, and on-call library support staff, with libraries located in the main hospitals and a specialty cancer library. We also managed the speaker’s bureau and published a monthly consumer health newsletter. By contrast, the JCL side had only one librarian splitting her time to support two campuses. During the affiliation process, CME duties were also added to her job.

The libraries reported to marketing (SHC) and medical staffing (JCL), but because marketing was so busy with the bigger system merger, the libraries were assigned to work on our business plan with one of the directors of the research institute. We built our business case on 4 sets of data: reviewing contracts and resources for duplication, performing a library user survey at both organizations, benchmarking against other hospital systems with a comparative market analysis of nonprofit teaching hospital systems of a similar post-merger size (over 1,000 beds), and surveying the reporting structure for hospitals that met the post-merger size criterion. In reviewing contracts, we were able to eliminate duplicate resources for a cost savings of $131,000. We benchmarked against 13 systems (4 local and 9 regional or national) with libraries that averaged 1 full-time staff person per library, with a focus on clinicians. Libraries were found that reported to medical education, medical staffing, research, or administration and none were found that reported to marketing. When we sent a survey to 540 clinician library users at both organizations, we received a 78% response rate, demonstrating tremendous clinician support favoring a library on each campus and, more importantly, a librarian at each hospital.

After the 2015 merger, all of our existing library spaces remained intact, our staffing was set for five master’s degree–prepared librarians (one per campus), CME was eliminated as a job duty, and we began reporting to medical education rather than marketing. We shifted our focus to working primarily with clinicians, and the speaker’s bureau and newsletter were transitioned out of the libraries and back to marketing.

We recently had some changes in the last few months with reductions throughout the hospital. Our manager retired, and I moved into the supervisor position, but my job as senior librarian has not been filled. My position now supervises libraries and the CME Department. We report to the chief academic officer, who is strategizing to help us impart our value. She is working on a “Learning Center” model—which will include new library space, a simulation lab, and collaborative meeting space—and has had us pilot what we call “librarians on the go,” with librarians spending less time at physical library desks and more time embedded in key high-visibility areas of the hospital, all with the hope that these steps will help us stabilize and eventually grow.

## DISCUSSION

In the audience question-and-answer period that followed the presentations, one librarian in attendance gave a brief explanation of her own recent situation that resulted in the loss of four librarian positions, including hers, and the closing of her library. She emphasized that her librarian skills are highly regarded and led to her hire in a nonlibrary faculty position teaching research and informatics. The rest of the audience discussion did not specifically address the case presentations but brought up general concerns and ideas about ways to prove value, the question of public librarians being seen as a threat or as collaborators, new library school graduates’ job anxiety, library school faculty teaching requirements and specialization tracks, and the need for an MLA marketing campaign similar to that launched by the American Library Association in 2017.

The library M&M case reports provide a collection of valuable, personal perspectives. These stories provide some insight into what it means to navigate a closure or reorganization. They also present the fear of knowing reorganization can mean losing a job or a library and the shock of learning about these decisions. Librarians who remained in their positions after a hospital library system merger found that their workloads increased. Health care providers in facilities that lost an on-site librarian might not receive information or guidance about where they should turn for library services. After the termination of a library service contract, librarians no longer employed by a hospital might continue to work for and help their former patrons.

Academic faculty librarians who serve as hospital librarians exemplify an alternative business model. Academic-affiliated library mergers are similar to hospital system library mergers in that they offer an opportunity to maintain hospital library services and access to a librarian, but in some cases, at the cost of keeping more librarians employed to maintain onsite library services with full-time librarian expertise. One of the cases described how a survey provided evidence that clinicians favored on-site hospital librarians and libraries. The librarian skill set is an asset to any organization and patient care team, but the library merger business model will continue to offer an attractive, cost-saving alternative.

The faculty librarians working under affiliation agreements to provide library services in a hospital setting both spoke about health care providers’ unequal access to information resources in a single institution or system due to providers’ academic affiliation or lack of one. An increase in the number of academic-affiliated providers in community hospitals was also seen to contribute to a decreasing need for hospital librarian services, especially in a health care system with system-wide information resources. However, unequal access to information resources also created an environment of information sharing and was seen as an opportunity for team-building between academic and community providers and the academic-affiliated librarian.

These stories show varying degrees of leadership through thoughtful planning and staff buy-in or indicate where the librarian was kept out of planning. In one merger case, an exemplary process for merger planning with librarian involvement and leadership was presented. However, even with a well-defined plan with librarian involvement, additional position losses were noted. In other cases, librarian involvement in planning appeared to be minimal, and it was left up to remaining staff to manage the consequences of library site closures and eliminated positions.

Factors that appear beyond librarians’ control, such as the economy and fluctuations in the competitive health care industry, also create continual stresses that impact non-revenue-producing departments such as libraries. Lastly, hope was not entirely lost, as it was noted that occasionally new libraries do open and open positions are being filled. Furthermore, at least in the panel cohort, the librarians who lost their jobs all found new ones.

The panel presentation experience leads to some generalizable research needs and advocacy goals. An accurate accounting of US health sciences libraries, including how many are staffed by librarians, is needed. This could be a collaborative effort with the American Libraries Directory, whose annual survey data are used by the *Library and Book Trade Almanac,* or the American Hospital Association, which has included a question about libraries in past surveys [14]. An association-administered procedure for reporting library closings could be helpful for future research. Collecting retrospective information on recently closed libraries is also warranted. Tracked data could be used to study whether loss of librarian services affects provider satisfaction, patient care, and health care quality indicators.

How do hospitals with librarians compare in patient care and other quality indicators against hospitals without librarians? Presenting and publishing about new library service delivery models and collaborations that maintain access to a librarian or best practices for centralizing library services in a health care system will also continue to benefit the profession. Finally, library and hospital leadership should involve hospital librarians in planning and implementing closures and consolidations. We may not always like management decisions, but we are experienced professionals who can be relied on to do what is best for our providers and patients.

## SUPPLEMENTAL FILE

Appendix Case Conference Outline: Navigating the Waters of Hospital Library Mergers, Crossing the Canyon of Hospital Library Closures: An M&M (Mortality & Mergers) Case Conference
