# Supplementary material for: Hospital library closures and consolidations: a case series
Source: J Med Libr Assoc. 2019 Apr 1;107(2):129–36. doi: 10.5195/jmla.2019.520 (PMC6466508; doi:10.5195/jmla.2019.520)
Supplement: Appendix [file jmla-107-129-s001.pdf]

JMLA Journal of the Medical Library Association

SC

Supplemental content to
J Med Libr Assoc. Apr;107(2):dx.doi.org/10.5195/jmla.2019.520
www.jmla.mlanet.org
© Harrow, Marks, Schneider, Lyubechansky, Aaronson, Kysh, Harrington 2019

Hospital library closures and consolidations: a case series

Andrea Harrow, MLS, AHIP; Lisa Marks, MLS, AHIP; Debra Schneider, MLIS, MEd; Alexander Lyubechansky, MA, MLIS; Ellen Aaronson, MLS, AHIP; Lynn Kysh, MLIS; Molly Harrington, MLS

APPENDIX

Case Conference Outline: Navigating the Waters of Hospital Library Mergers, Crossing the Canyon of Hospital Library Closures: An M&M (Mortality & Mergers) Case Conference

Statement of Need: Statistical data from the Task Force on Vital Pathways for Hospital Librarians [1] and literature review reveals that hospital libraries are being closed or are under threat of closure, but there is very little published that looks at context and decision process or the effect of these changes on health care provider satisfaction or patient outcomes. Preventing closure is not always possible, but understanding how these decisions are made and carried out could also inform best practice management and planning of a consolidation or closure.

A panel of four to six librarians will tell their stories of navigating a library closure or reorganization through case presentation. Background information and context will highlight reasons the decisions to reorganize or close was made. Following the case presentations, participants and attendees will take part in Q&A and discussion. What can we learn from these closings and mergers? Were library services maintained; what services were changed? Does this tell us anything new about changing library models? How did librarians who lost their jobs recover or adapt? During the discussion, presenters will assess and discuss their library closings or reorganizations. Together with the participating audience, presenters will generate collaborative ideas to possibly manage these crises with maintained services. Cases and discussion points will be recorded and communicated to the Hospital Libraries Section for further discussion, research, publication, and advocacy.

Format: Panel presentation with discussion/Q&A to follow. Panelists will each have five minutes to present their cases to include:

1. Introduction or Title: One or two sentences summarizing the case.
2. Case Presentation: Explicit background, try and cover all the factors that led to the decision to close or consolidate, if known. Use a timeline, if helpful.
3. Management and Outcome: Describe your role in the consolidation or shut-down. What did you advise your clientele? Do you know where providers, staff, and patients now go for library services and access to evidence-based information and if they are satisfied? What effect has the reorganization or closing of the library had on the hospital community? Were there benefits gained or adverse effects experienced with the reorganization or closing of the library? Were library services reinstituted in another form?
4. Assessment and Discussion: Given the circumstances of the closing or reorganization, could it have been handled differently? Could you have done anything differently? What is your advice to librarians going through a similar situation? Where are you now?

After presentations, moderator will facilitate discussion involving Q&A from attendees and reference to the medical literature. Advocacy ideas and future direction will be formulated.

REFERENCE

1. Thibodeau PL, Funk CJ. Trends in hospital librarianship and hospital library services: 1989 to 2006. J Med Libr Assoc. 2009 Oct;97(4):273–9. DOI: http://dx.doi.org/10.3163/1536-5050.97.4.011.
